# Annual of the Universal Medical Sciences

**Published:** 1890-09

**Authors:** 


*Annual of the Universal Medical Sciences*. Edited by CHARLES E. SAJOUS, M.D. Five volumes. Philadelphia: F. A. DAVIS. 1890.

It is a genuine pleasure to record that this stupendous work, now in its third year, maintains the high character of the previous issues, and indeed in some parts surpasses it. The volumes for this year were somewhat delayed, owing to the fact that many of the editor's co-workers were attacked with influenza; but Dr. Sajous is to be congratulated on the way in which he overcame all his difficulties, and upon the co-operation of printers and illustrators who have all combined to present an attractive summary of recent medical work in all its branches. Without this publication, a man must be practising his profession at a disadvantage.

With each succeeding year the work is undergoing improvements more and more in the direction of rendering it of the greatest possible practical service; and its value is considerably enhanced by the numerous and excellent illustrations, coloured and uncoloured, with which it abounds.

The profession is under great obligation to Dr. Sajous and his helpers.

